# Radioactivity and Space Range of Ultra-Low-Activity for *in vivo* Off-line PET Verification of Proton and Carbon Ion Beam—A Phantom Study

**DOI:** 10.3389/fpubh.2021.771017

**Published:** 2021-12-06

**Authors:** Fuquan Zhang, Junyu Zhang, Yan Lu, Yixiangzi Sheng, Yun Sun, Jiangang Zhang, Jingyi Cheng, Rong Zhou

**Affiliations:** ^1^College of Physics, Sichuan University, Chengdu, China; ^2^Shanghai Key Laboratory of Radiation Oncology, Shanghai, China; ^3^Department of Nuclear Medicine, Shanghai Proton and Heavy Ion Center, Fudan University Cancer Hospital, Shanghai, China; ^4^Shanghai Engineering Research Center of Proton and Heavy Ion Radiation Therapy, Shanghai, China; ^5^Department of Radiotherapy, Shanghai Proton and Heavy Ion Center (SPHIC), Shanghai, China

**Keywords:** ultra-low activity, off-line PET, proton therapy, beam range, PET verification

## Abstract

**Purpose:** The radioactivity induced by proton and heavy ion beam belongs to the ultra-low-activity (ULA). Therefore, the radioactivity and space range of commercial off-line positron emission tomography (PET) acquisition based on ULA should be evaluated accurately to guarantee the reliability of clinical verification. The purpose of this study is to quantify the radioactivity and space range of off-line PET acquisition by simulating the ULA triggered by proton and heavy ion beam.

**Methods:** PET equipment validation phantom and low activity ^18^F-FDG were used to simulate the ULA with radioactivity of 11.1–1480 Bq/mL. The radioactivity of ULA was evaluated by comparing the radioactivity in the images with the values calculated from the decay function with a radioactivity error tolerance of 5%. The space range of ULA was evaluated by comparing the width of the R50 analyzed activity distribution curve with the actual width of the container with a space range error tolerance of 4 mm.

**Results:** When radioactivity of ULA was >148 Bq/mL, the radioactivity error was <5%. When radioactivity of ULA was >30 Bq/mL, the space range error was below 4 mm.

**Conclusions:** Off-line PET can be used to quantify the radioactivity of proton and heavy ion beam when the ULA exceeds 148 Bq/mL, both in radioactivity and in space range.

## Introduction

*In vivo* biological verification using positron emission tomography (PET) is one of the most important estimation methods in proton or heavy ion precision radiotherapy (1–4). Models of *in vivo* biological verification using PET can be classified into three types: in-beam, in-room and off-line. In-beam and off-line methods are most frequently used in research studies and clinical practice to evaluate the precision of proton or heavy ion beam. The in-beam PET is an ensemble of PET and particle radiotherapy terminals that can gather the β+ signal throughout particle beam delivery. In-beam imaging is little influenced by human metabolism and blood flow, and it can increase measurement accuracy (5–7). In-room PET uses a stand-alone and full-ring PET scanner positioned in the treatment room to scan the patient (still in the treatment bed) soon after treatment. In-room PET is a compromise between in-beam and off-line PET (8). The off-line PET is more applicable: patients are transferred to the PET/CT equipment room for gathering of the β+ signal after particle beam delivery (9, 10).

Compared with the in-beam PET, the off-line PET has several advantages such as much lower cost, shorter treatment time, and increased suitability for clinical practice (11, 12). Off-line PET offers a practical and easy-to-implement method of treatment verification for particle radiotherapy centers with PET/CT scanners located near their treatment rooms. In particle therapy, the detectable activation results from nuclear fragmentation reactions between the projectiles and the target nuclei of the traversed tissue. Proton-induced radioactivity is thus very sensitive to the elemental composition. These sources of uncertainty are reduced in the off-line scenario because of the small number of production channels that yield long-lived positron emitters (13, 14). Because of these two prominent advantages, off-line PET has achieved wide recognition in clinical practice.

In practice, the β+ signal of acquisition in off-line PET is mainly emitted by ^11^C (20.39 min). However, there is a 10-min interval between beam delivery and PET acquisition, which can also cause large reductions in radioactivity. PET image quality (radioactivity and beam range) is compromised when the interval time of off-line PET is too long. The radioactivity in the tumor of off-line PET imaging is 37–370 Bq/mL, which is far below the level of conventional ^18^FDG (fluorodeoxyglucose) PET/CT imaging (over 7,400 Bq/mL) (15). In clinical practice, the mean radioactivity of 289 Bq/mL in proton radiotherapy for breast cancer has been obtained by calculating the radioactivity of each spot within the target area. The average radioactivity of carbon ion radiotherapy in the anterior gland, liver and head tumors were 90.65, 109.89, and 138.75 Bq/mL, respectively. Hence, whether radioactivity and space range of off-line PET at ultra-low-activity (ULA) is reliable needs further verification.

The radioactivity and space range of commercial off-line PET acquisitions based on ULA should be evaluated accurately to guarantee the reliability of clinical verification. The purpose of this study is to quantify the radioactivity and space range of off-line PET acquisition by simulating the ULA triggered by proton and heavy ion beam using verification phantom.

## Materials and Methods

### Equipment and Verification Phantom

The PET-CT device used in this research was a Biograph mCT PET/CT scanner (Siemens Medical Solutions USA), which has four rings of 192 blocks in total, each of which contains 13 × 13 lutetium-oxyorthosilicate (LSO) crystals with dimensions of 4 × 4 × 20 mm. The voltages of the X-ray tube in CT were 80, 100, 120, and 140 kV, respectively. The planar resolution of the reconstruction image was 4 × 4 mm, and its thickness was 0.6 mm. The PET detection system had four detection rings, each of which contained 48 detection blocks. Each detection block was uniformly divided into 13 × 13 basic detection units, and the crystal size of each detection unit was 4 × 4 × 20 mm. The aperture of the detector was 78 cm and the field of view of the detector's axial was 21.8 cm. The detector's gating window was 4.1 ns and its energy window was 435–650 keV. We used the Truex image reconstruction algorithm which incorporates OSEM (Ordered Subsets Expectation) iterative algorithm and point-spread-function correction. CT attenuation correction is used in image reconstruction.

**Figure 1:**
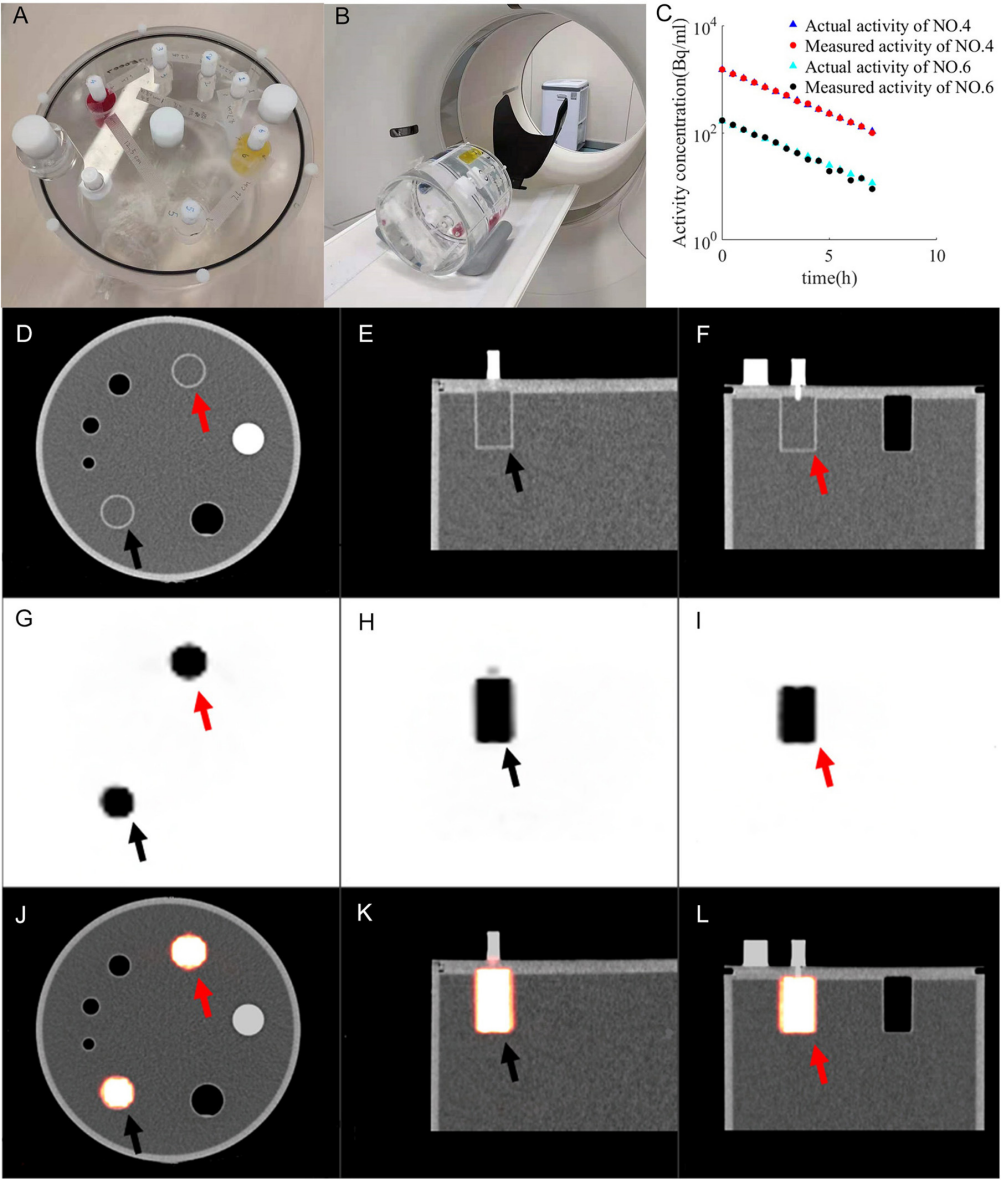
Imaging of PET validation phantom **(A)** under PET/CT **(B)**. CT images **(D–F)** and PET images **(G–I)** form fused images **(J–L)**. The activity of container 4 (red arrow) and container 6 (black arrow) changes with time **(C)**. *R*_*actu*_ of container 4 (blue triangle), *R*_*meas*_ of container 4 (red dot), *R*_*actu*_ of container 6 (green triangles), *R*_*meas* of container 6 (black dot) are distributed between 11.1 and 1,480 Bq/mL.

Using a PET validation phantom (Flanged Jaszczak ECT Phantom), referring to the National Electrical Manufacturers Association (NEMA) standard, we simulated the ULA of the target after beam delivery. The phantom was cylindrical in shape, its external height was 22.24 cm, its bottom diameter was 19.24 cm, its wall thickness was 3.2 mm, and the material was PTFE. As shown in Figure 1A, six cylindrical containers of the same height were arranged in a ring inside the phantom. Their diameters were 8 mm (No. 1), 12 mm (No. 2), 16 mm (No. 3), and 25 mm (Nos. 4, 5, and 6), and their height was 38.1 mm. Thus, the cylinders' volumes were 1.92 ml (No. 1), 4.31 ml (No. 2), 7.66 ml (No. 3), and 18.70 ml (Nos. 4, 5, and 6). During the loading of radiopharmaceuticals into the container, small bubbles often appeared inside the small containers, so we chose the larger containers. The containers (Nos. 4 and 6) were selected as the research objects because of their large volumes and reduced bubble effects.

### Experimental Design

The ULA of the experimental design was 11.1–1,480 Bq/mL. Smaller radioactivity would lead to a greater error when measuring activity. Because the measurement error of high-activity radiopharmaceuticals is small, to obtain accurate values for the activity during the measurements, the active compounds were filled into the containers 10 half-lives in advance. The radioactivity of radiopharmaceuticals was measured by the radio activity meter CRC-25R with resolution of 0.01 MBq. The radiopharmaceutical (^18^F-FDG) at the dose of 27.09 MBq was loaded into each container (Nos. 4 and 2). The radiopharmaceutical at the dose of 95 MBq was loaded into container No. 6 with an error rate lower than one-thousandth. Then, the dye is injected into the container. After freezing, the dye and solution became solid. The container was subsequently full filled with distilled water. The interval between charging radioactive radiopharmaceuticals and scanning is 10 half-lives.

In this experiment, to account for attenuation correction, the phantom was filled with water. PET acquisition was initiated when the radioactivity in the container reached the expected range of radioactivity (Figure 1B). The selected parameters were weight of 50 kg, dose of 10 mCi, reconstruction layer thickness of 5 mm and Na element collection for acquisition (16). The decay parameter was not added in the process of PET reconstruction because the half-life of ^22^Na is 2 years. Therefore, we set the acquisition method as Na-22 to avoid any decay correction. The phantom was positioned on the scanner couch and did not moved during acquisitions. Each acquisition time was 15 min, the acquisition interval was 15 min, the total number of scans was 15, and the total acquisition time was 8 h.

### ULA Was Quantitatively Analyzed Based on Comparing Measured and Actual Radioactivity

The radioactivity in the containers was evaluated from the PET images; the methods are according to the half-life formula:


$$\begin{array}{l} A=A_{0}{\left(\frac{1}{2}\right)}^{\frac{t}{T}} \end{array}$$

where A_0_ is the radioactivity before decay, the t is the decay time, and T is the half-life. The variable A would express the activity at time t.

There are two ways to measure the radioactivity in the container: (1) measurement of the radioactivity of the whole container, and then dividing it by the volume of the container to calculate the radioactivity; (2) The radioactivity distributed at the points inside the container (at points above 5 mm from the container wall) was sampled and measured. These two methods are used, respectively, to measure radioactivity.

Comparing the measured radioactivity (*R*_*meas*_) with the actual radioactivity (*R*_*actu*_), *R*_*actu*_ was calculated from the decay formula. *R*_*meas*_ is compared *R*_*actu*_ to obtain the error value according to the following formula:


$$\begin{array}{l} \text{error}=\frac{R_{actu}-R_{meas}}{R_{actu}} \end{array}$$

An appropriate function of radioactivity was obtained by fitting the error rate, from which the error rate (and its confidence interval) could be calculated. In this study, the measurement error of the radioactivity obtained from the PET images was allowed to be <5% according to the report of The American Association of Physicists in Medicine (AAPM) TG 126 (17).

### Space Range of ULA Was Quantitatively Analyzed Using the Method of R50

**Figure 2:**
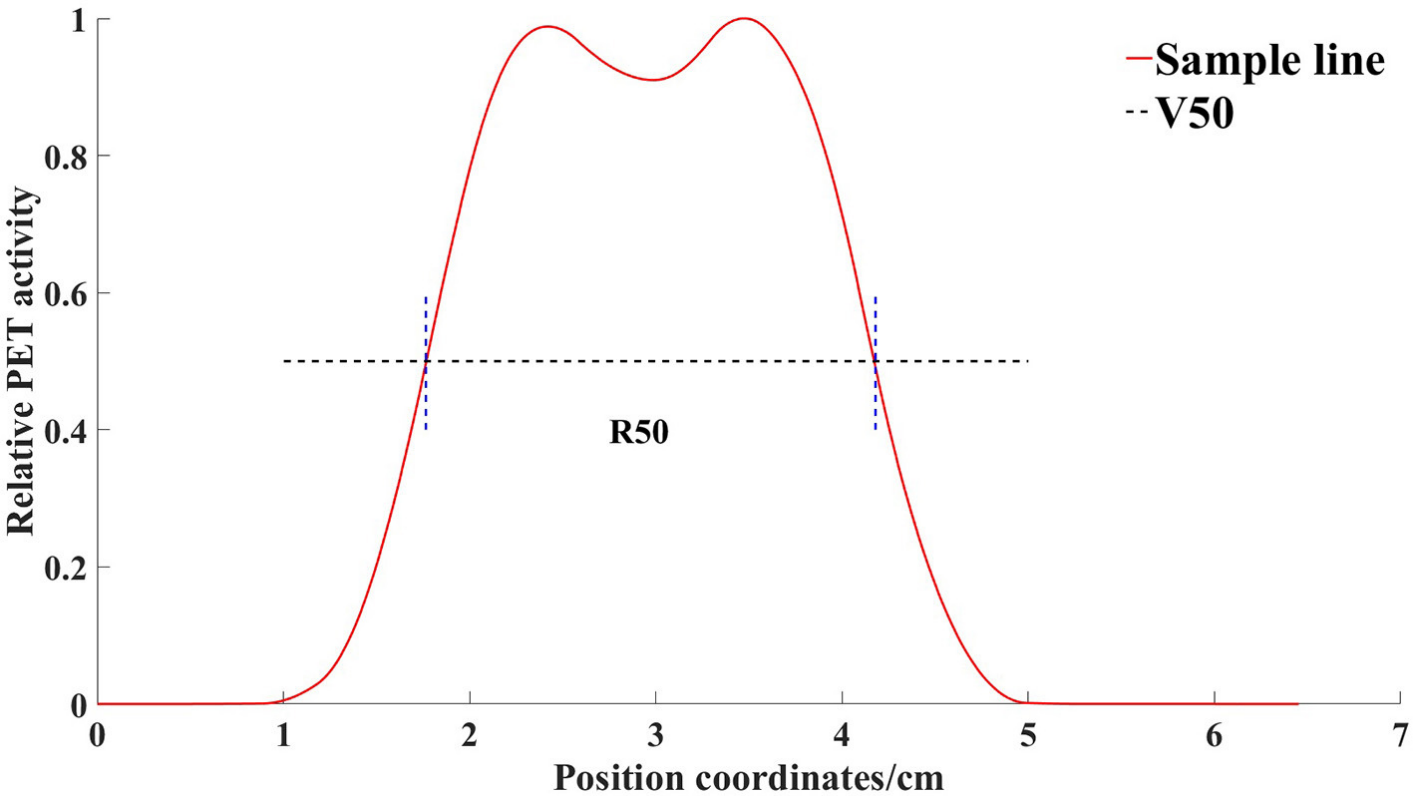
Radioactivity distribution curve of a line through the center of the container obtained by the sampling line. V50 is the horizontal line where half of the maximum value is located. R50 refers to the distance between the points where the anterior and posterior two functional values are equal to half of the peaks, in one peak of the function.

First, we marked the center points of cylinders Nos. 4 and 6 on each layer of the PET-CT images. Then, across the center of the circle and along the X-axis and Y-axis, we created sampling lines to obtain the radioactivity values of the PET image on their paths. We used the R_50_ method (17) to analyze the width of the radioactivity distribution curve (Figure 2). R50 refers to the distance between the points where the anterior and posterior two functional values are equal to half of the peaks, in one peak of the function.

An appropriate function of radioactivity was obtained by fitting the width error, from which the width error (and its confidence interval) could be calculated. According to the report of AAPM Task Group 126 (17), the allowable error in PET/CT joint registration is ±1 PET voxel, meaning that a 4-mm error is allowed in the space range.

## Results

### The Model of ULA Was Built Using the Validation Phantom

The change in radioactivity throughout the entire acquisition time is shown in Figure 1C. *R*_*actu*_ of container 4 (blue triangle), *R*_*meas*_ of container 4 (red dot), *R*_*actu*_ of container 6 (green triangles) and *R*_*meas*_ of container 6 (black dot) are distributed between 11.1 and 1,480 Bq/mL. *R*_*actu*_ is the actual radioactivity. *R*_*meas*_ is the measured radioactivity. The resulting PET-CT image is shown in Figures 1D–L. CT images (Figures 1D–F) and PET images (Figures 1G–I) form fused images (Figures 1J–L).

### Quantitative Results of ULA

**Figure 3:**
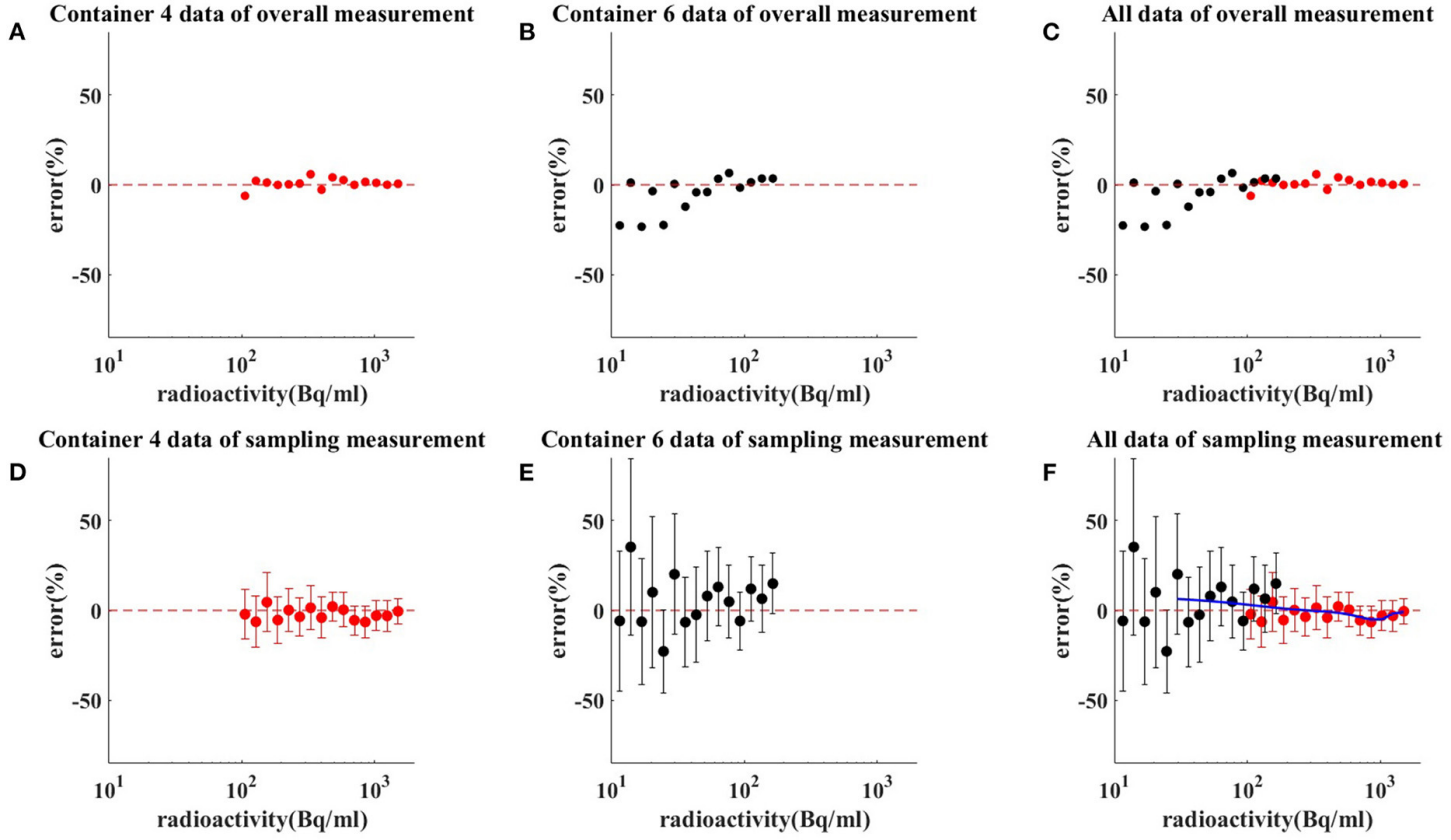
Results of measuring radioactivity by method 1 **(C)** and method 2 **(F)**. Red dot and red bar represent the mean radioactivity error and the standard error of container 4 **(A,D)**, respectively. Black dot and black bar represent the mean radioactivity error and the standard error of container 6 **(B,E)**, respectively. When radioactivity between 11.1–111, 111–370, and 370–1,480 Bq/mL, the mean activity errors of the overall measurement were 8.83, 2.04, and 1.45%, respectively. When radioactivity between 11.1–111, 111–370, and 370–1,480 Bq/mL, the mean activity errors of sampling measurement were 4.1 ± 26.43, 2.69 ± 14.6, and 1.60 ± 8.73%, respectively. The blue line is the radioactivity error fitted curve for ULA.

Both method 1 (overall measurements) and method 2 (sampling measurements) analyzed PET images for activity values. Depending on measured radioactivity at drug loading, time and decay formula, radioactivity error at different radioactivity from container 4–6 is calculated. The comparison results between the *R*_*meas*_ and *R*_*pred*_ by method 1 are shown in Figures 3A–C. The comparison results between the *R*_*meas*_ and *R*_*pred*_ by method 2 are shown in Figures 3D–F. The results of the measure radioactivity error of the overall and sampling measurement are shown in Table 1. When radioactivity between 11.1–111, 111–370, and 370–1,480 Bq/mL, the mean activity errors of the overall measurement were 8.83, 2.04, and 1.45%, respectively, and the mean activity errors of sampling measurement were 4.1 ± 26.43, 2.69 ± 14.6, and 1.60 ± 8.73%, respectively. An appropriate function of radioactivity was obtained by fitting the radioactivity error rate, which was calculated to be 2.81%, with a confidence interval of [1.09–4.54%] at 148 Bq/ml. When radioactivity of ULA was >148 Bq/mL, the radioactivity error was <5%.

**Table 1:** Radioactivity errors between measured radioactivity and actual radioactivity.

**Actual radioactivity concentration (Bq/mL)**	**Radioactivity errors of overall container measurements (%)**	**Radioactivity errors of sampling measurements (%)**
11.1–111	8.83	4.1 ± 26.43
111–370	2.04	2.69 ± 14.6
370–1,480	1.45	1.60 ± 8.73

### Quantitative Results of ULA Space Range

**Figure 4:**
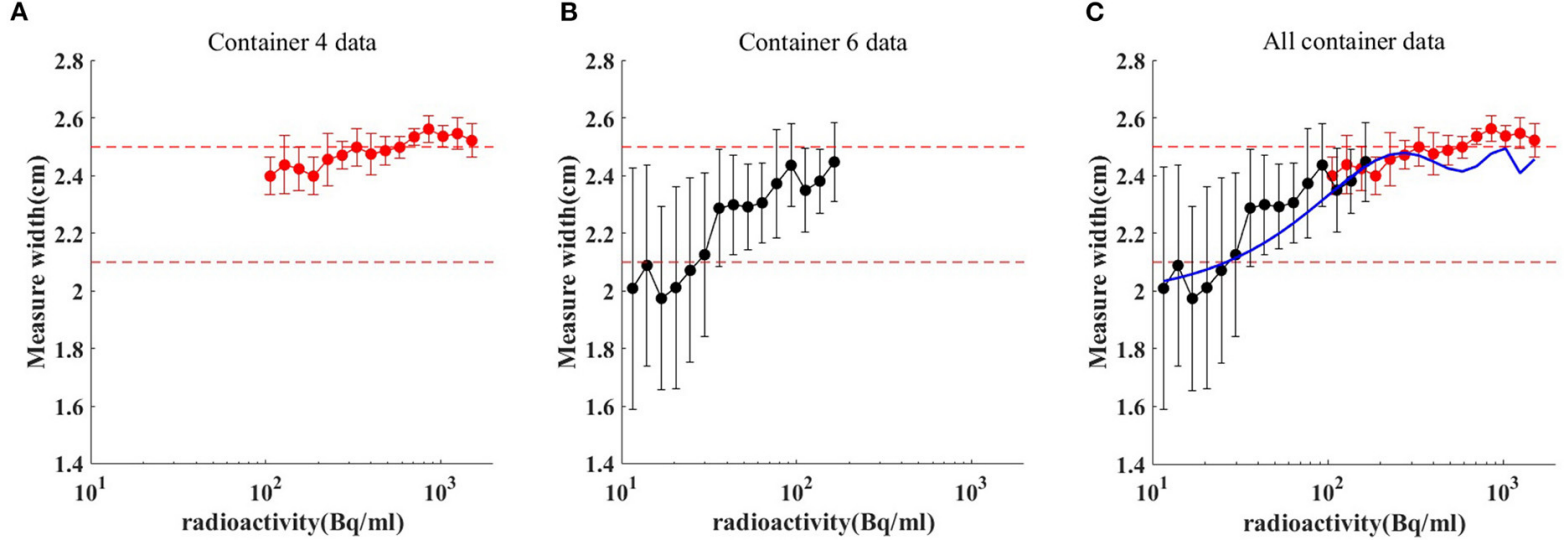
Comparison of container width measured by R50 method **(C)** with actual. Red dot and red bar represent the mean width and the standard deviation of container 4 **(A)**, respectively. Black dot and black bar represent the mean width and the standard deviation of container 6 **(B)**, respectively. When radioactivity between 11.1–111, 111–370, and 370–1,480 Bq/mL, the average width was 2.189 ± 0.253, 2.426 ± 0.09, and 2.521 ± 0.047 cm, respectively. The blue line is the radioactivity width error fitted curve for ULA.

The spatial range reliability verification analysis was performed using the R50 method (i.e., active depth curve) to compare container widths. The results are shown in Figure 4. Comparison of container width measured by R50 method with actual width is shown in Table 2. When radioactivity between 11.1–111, 111–370, and 370–1,480 Bq/mL, the average width was 2.189 ± 0.253, 2.426 ± 0.09, and 2.521 ± 0.047 cm, respectively. An appropriate function of radioactivity was obtained by fitting the width error. When radioactivity of ULA was 30 Bq/mL, the width error was 3.8 mm [3.66–3.97 mm]. When radioactivity of ULA was 148 Bq/mL, the width error was 0.87 mm [0.84–0.90 mm]. When radioactivity of ULA was 259 Bq/mL, the width error was 0.3 mm [0.12–0.48 mm]. When radioactivity of ULA was >30 Bq/mL, the width error was below 4 mm.

**Table 2:** Comparison of container width measured by R50 method with actual width (2.5 cm).

**Actual radioactivity concentration (Bq/mL)**	**Average value of width measured (cm)**	**Error between average width and actual width**
11.1–111	2.189 ± 0.253	−3.10 ± 0.253
111–370	2.426 ± 0.091	−0.73 ± 0.091
370–1,480	2.521 ± 0.047	0.21 ± 0.047

## Discussion

The material of the quality control phantom (Flanged Jaszczak ECT the Phantom) is PMMA, which top consists are six containers and a cylindrical PMMA, and the resolution module can be placed inside. The NEMA PET quality control phantom used in this research is one of the most widely accepted standards, which can provide a variety of measurement methods for the quality control of PET scanning. Standards for quality control models were developed with reference to the requirements of PET by many institutions, including the Society of Nuclear Medicine and Molecular Imaging (SNMMI), International Electrotechnical Commission (IEC), International Atomic Energy Agency (IAEA), American College of Radiology (ACR), and National Electrical Manufacturers Association (NEMA) (18).

In this study, the ULA of off-line PET was investigated in terms of both radioactivity and space range. When radioactivity of ULA was >148 Bq/mL, the radioactivity error was <5%. This indicates that the off-line PET can meet the radioactivity requirement when the ULA exceeds 148 Bq/mL. When radioactivity of ULA was >30 Bq/mL, the space range error was below 4 mm. This indicates that the off-line PET can meet the space range requirement when the ULA exceeds 30 Bq/mL. Our study complements previous work on off-line PET.

To the best of our knowledge, there have been no reports on quality control for the ULA of off-line PET. Bauer et al. (19) proves the feasibility of the implemented strategy for offline confirmation of scanned carbon ion irradiation. On this basis, Knopf et al. (14) evaluated the impact of the following aspects on the feasibility and accuracy of the off-line PET/CT method by Monte Carlo: (1) biological washout procedure, (2) patient motion, (3) tissue classification based on Hounsfield units (HU) for simulating activity distribution, and (4) tumor site specificity. But the assessment of the reliability of PET imaging at ULA is missing.

Spacer range was set for parameters of beam range and depth verification *in vivo* PET verification (16, 20). Parodi et al. (6, 16) suggested that beam range could be verified within an accuracy of 1–2 mm in head off-line proton verification. Zhang et al. (21) investigated the feasibility of depth verification of off-line PET/CT treatment verification in phantom. The mean radioactivity of 289 Bq/mL in proton radiotherapy for breast cancer has been obtained by calculating the radioactivity of each spot within the target area. When radioactivity of ULA was 259 Bq/mL, the width error was 0.3 mm [0.12–0.48 mm]. This represents that ULA may result in a 0.3 mm error for breast cancer proton off-line PET verification.

Slight errors may lead to unreliable results when operating at low radioactivity, so the accurate filling of quality control phantom determines its reliability for testing. This problem was solved by filling the phantom with radiopharmaceuticals 10 h in advance. The radioactivity was still high when the radiopharmaceuticals were loaded, so the measurement error can be ignored. The position of the quality control phantom was fixed, and the continuous acquisition for a long period ensured not only location registration but also the continuity of the data. The assessment of radioactivity was greatly affected by the leakage of liquid. In practical operation, liquid leakage is very easy to occur in the process of removing air bubbles. Therefore, we used dye in solution to observe if leakage of fluid occurred. A method of freezing radiopharmaceuticals was used to reduce the effect caused by leakage of fluid during dilution. Therefore, we could solve the problem of radioactive liquid leakage by freezing and dyeing, which could facilitate more accurate comparisons of the radioactive values.

## Conclusions

PET equipment validation phantom with ulter- low activity 18F-FDG can used to simulate the radioactivity of ULA. When radioactivity of ULA was >148 Bq/mL, the radioactivity error was <5%. When radioactivity of ULA was >30 Bq/mL, the space range error was below 4 mm. Off-line PET can be used to quantify the radioactivity based on proton and heavy ion beam when the ULA exceeds 148 Bq/mL, both in radioactivity and in space range.

## Data Availability Statement

The original contributions presented in the study are included in the article/supplementary material, further inquiries can be directed to the corresponding author/s.

## Author Contributions

JuZ and YL contributed to the conception of the study. JuZ and YSh contributed significantly to analysis and manuscript preparation. FZ performed the data analyses and wrote the manuscript. JiZ and YSu helped perform the analysis with constructive discussions. RZ and JC are responsible for ensuring that the descriptions are accurate and agreed by all authors. All authors contributed to the article and approved the submitted version.

## Conflict of Interest

The authors declare that the research was conducted in the absence of any commercial or financial relationships that could be construed as a potential conflict of interest.

## Publisher's Note

All claims expressed in this article are solely those of the authors and do not necessarily represent those of their affiliated organizations, or those of the publisher, the editors and the reviewers. Any product that may be evaluated in this article, or claim that may be made by its manufacturer, is not guaranteed or endorsed by the publisher.
