# Corrigendum: Radioactivity and space range of ultra-low-activity for *in vivo* off-line PET verification of proton and carbon ion beam—a phantom study

**DOI:** 10.3389/fpubh.2024.1505079

**Published:** 2024-10-22

**Authors:** Fuquan Zhang, Junyu Zhang, Yan Lu, Yixiangzi Sheng, Yun Sun, Jiangang Zhang, Jingyi Cheng, Rong Zhou

**Affiliations:** ^1^College of Physics, Sichuan University, Chengdu, China; ^2^Shanghai Key Laboratory of Radiation Oncology, Shanghai, China; ^3^Department of Nuclear Medicine, Shanghai Proton and Heavy Ion Center, Fudan University Cancer Hospital, Shanghai, China; ^4^Shanghai Engineering Research Center of Proton and Heavy Ion Radiation Therapy, Shanghai, China; ^5^Department of Radiotherapy, Shanghai Proton and Heavy Ion Center (SPHIC), Shanghai, China

**Keywords:** ultra-low activity, off-line PET, proton therapy, beam range, PET verification

In the published article, there was an error in the Acknowledgments statement. The grant number for Natural Science Foundation of Shanghai was incorrect—the correct number is “21ZR1460300.” The correct Acknowledgments statement appears below.

